# Prognostic Impact of Malnutrition Evaluated via Bioelectrical Impedance Vector Analysis (BIVA) in Acute Ischemic Stroke: Findings from an Inverse Probability Weighting Analysis

**DOI:** 10.3390/nu17050919

**Published:** 2025-03-06

**Authors:** Simone Dal Bello, Laura Ceccarelli, Yan Tereshko, Gian Luigi Gigli, Lucio D’Anna, Mariarosaria Valente, Giovanni Merlino

**Affiliations:** 1 Clinical Neurology Unit, Santa Maria della Misericordia University Hospital, 33100 Udine, Italy; 2 Department of Medical Area, University of Udine, 33100 Udine, Italy; 3 SOSD Stroke Unit, Department of Head-Neck and Neuroscience, Azienda Sanitaria Universitaria Friuli Centrale (ASUFC), 33100 Udine, Italy; 4 Department of Stroke and Neuroscience, Charing Cross Hospital, Imperial College Healthcare NHS Trust, London W2 1NY, UK; 5 Department of Brain Sciences, Imperial College London, London SW7 2AZ, UK

**Keywords:** BIA, BIVA, Bioelectrical Impedance Analysis, Bioelectrical Impedance Vector Analysis, ischemic stroke

## Abstract

**Background.** The association between malnutrition and poor outcomes in stroke patients has, to date, been evaluated using composite scores derived from laboratory measurements. However, Bioelectrical Impedance Analysis (BIA) and its advanced application, Bioelectrical Impedance Vector Analysis (BIVA), offer a non-invasive, cost-efficient, and rapid alternative. These methods enable precise assessment of body composition, nutritional status, and hydration levels, making them valuable tools in the clinical evaluation of stroke patients. **Objective.** This study aimed to compare the ordinal distribution of modified Rankin Scale (mRS) scores at 90 days following an acute ischemic stroke, stratifying patients based on their nutritional status at the time of Stroke Unit admission, as determined by the Bioelectrical Impedance Vector Analysis (BIVA) malnutrition parameter. **Methods.** We conducted a single-centre prospective observational study on all consecutive patients admitted for acute ischemic stroke to our Stroke Unit between 1 April 2024, and 30 September 2024. We applied the IPW (Inverse Probability Weighting) statistical technique and ordinal logistic regression to compare mRS scores in malnourished and non-malnourished patients. **Results.** Overall, our study included 195 patients with ischemic stroke assessed using BIVA. Of these, 37 patients (19%) were malnourished. After IPW, we found that malnourished patients had significantly lower rates of favorable 90-day functional outcomes (cOR 3.34, 95% CI 1.74–6.41; *p* = 0.001). Even after accounting for relevant covariates, malnutrition remained an independent predictor of unfavorable outcomes (acOR 2.79, 95% CI 1.37–5.70; *p* = 0.005), along with NIHSS score at admission (acOR 1.19, 95% CI 1.11–1.28; *p* < 0.001), intravenous thrombolysis (acOR 0.28, 95% CI 0.15–0.52; *p* < 0.001), absolute lymphocyte count (cOR 1.01, 95% CI 1.00–1.02; *p* = 0.027), and albumin concentration (cOR 0.82, 95% CI 0.75–0.89; *p* < 0.001). **Conclusions.** Malnutrition, assessed through Bioelectrical Impedance Vector Analysis (BIVA) at the time of admission to the Stroke Unit, is associated with worse clinical outcomes at 90 days following the ischemic cerebrovascular event.

## 1. Introduction

Stroke is one of the leading causes of morbidity and mortality in Western populations, placing a significant financial burden on healthcare systems worldwide [1]. Stroke is a heterogeneous and multifactorial disease, regulated by both modifiable and non-modifiable risk factors. Approximately 80% of stroke events could be prevented by making simple lifestyle modifications [1]. The correlation between malnutrition and poorer prognosis in stroke patients is well-documented in the literature [2,3].

According to ESPEN guidelines, malnutrition (or undernutrition) is defined as a condition caused by inadequate nutrient intake or absorption, leading to altered body composition—specifically, a reduction in lean and body cell mass. This results in diminished physical and mental function and compromised clinical outcomes due to disease [4].

Malnutrition is a serious issue with a negative impact on patients’ quality of life and clinical outcomes, contributing to increased morbidity, longer in-hospital stays, higher mortality rates, and rising healthcare expenses. Early identification is crucial for implementing necessary therapeutic actions, including appropriate nutritional support, to prevent or resolve malnutrition [5].

To date, malnutrition has primarily been analyzed using composite laboratory scores, such as the CONUT score, Geriatric Nutritional Risk Index (GNRI), Prognostic Nutritional Index (PNI), and HALP score (Hemoglobin, Albumin, Lymphocyte, and Platelet score) [6,7,8,9]. Although these composite laboratory scores are simple to apply and useful for detecting malnutrition and predicting long-term outcomes, they have certain limitations [10]. Specifically, composite scores rely on laboratory analyses, which come with associated costs and technical processing times. As a result, they do not allow for real-time nutritional assessment or personalized monitoring [6,7,8,9]. Even measurements such as BMI, skinfold thickness assessment, and abdominal circumference remain limited and problematic, often unable to provide an accurate evaluation—especially in conditions like normal weight obesity [11].

Bioelectrical Impedance Analysis (BIA) and Bioelectrical Impedance Vector Analysis (BIVA) are non-invasive, cost-effective, and rapid methods for accurately assessing body composition and hydration status in clinical practice [12]. These methods provide more precise data on altered body composition, such as a reduction in lean mass or changes in cellular mass. To date, these methods are widely used in various clinical fields, including oncology, nephrology, and cardiology [13,14,15].

The physical principle of BIA relies on the varying electrical conductivity of body tissues when exposed to an alternating microcurrent (800 μA at 50 kHz). As the current travels through the body, it encounters resistance (R) from bodily fluids and reactance (Xc) from cell membranes. These two parameters are measured using specialized devices with four surface electrodes placed on the hand and foot, allowing for accurate assessment of body composition and tissue status [16].

From the values of resistance (R) and reactance (Xc), impedance can be derived, typically expressed as the modulus |Z| = √(R^2^ + Xc^2^), along with the phase angle PhA = arctan(Xc/R) [17].

In clinical practice, regression analysis has been used to develop empirical equations that allow the use of bioimpedance values (R and Xc) to estimate key parameters of body composition. These include intracellular water (ICW), extracellular water (ECW), fat mass (FM), and fat-free mass (FFM) [16].

Although BIA offers numerous advantages for assessing body composition, it has certain limitations related to the equations used for calculations. The results can be influenced by various factors, including abnormalities in body shape, ethnic differences, extreme body mass index (BMI) values, and fluid imbalances [18].

BIVA, introduced by Piccoli et al. [19], represents an innovative evolution of traditional BIA. It was developed to assess nutritional and hydration status using a bivariate vector analysis that integrates reactance and resistance standardized by height, overcoming some limitations of conventional BIA. Unlike BIA, BIVA does not rely on regression equations or body weight to estimate body composition. By exclusively using raw impedance measurements, BIVA proves suitable for conditions involving significant weight or fluid volume variations [18].

The aim of the study is to evaluate whether ischemic stroke patients classified as malnourished according to BIVA parameters upon Stroke Unit admission have a worse clinical outcome compared to non-malnourished patients.

## 2. Materials and Methods

### 2.1. Study Design and Patients

**Figure 1:**
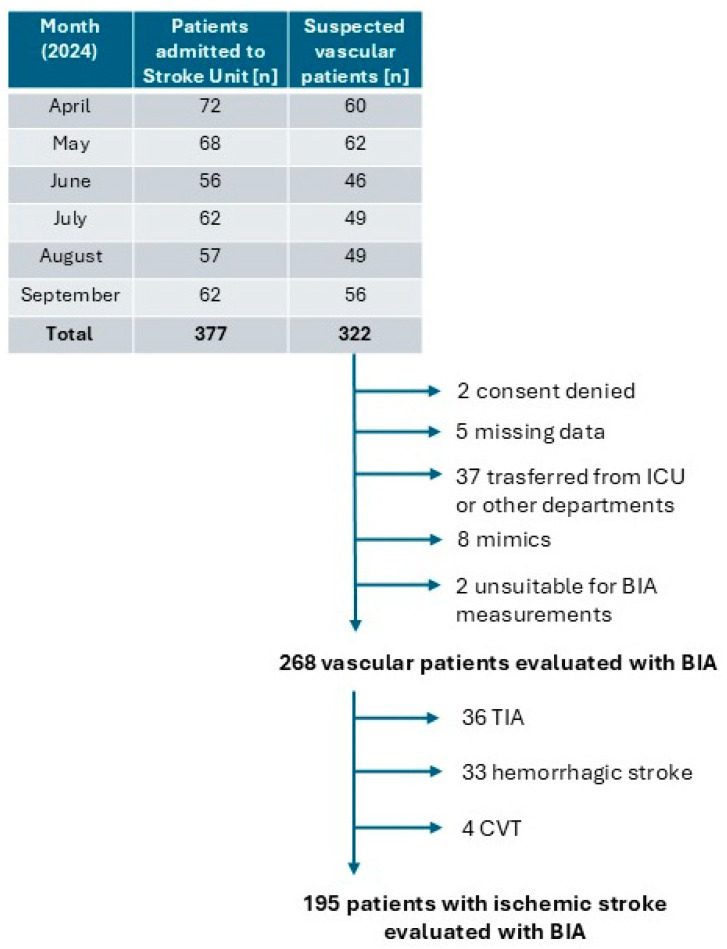
Flowchart of patients admitted to the Stroke Unit during the study period, assessed using the BIA method, and included or excluded from the study (Legend: ICU = intensive care unit; BIA = Bioelectrical Impedance Analysis; TIA = transient ischemic attack; CVT = cerebral venous thrombosis).

A single-center, prospective, cohort study was conducted on all Acute Ischemic Stroke (AIS) patients consecutively admitted to the Stroke Unit of the Azienda Sanitaria Universitaria Friuli Centrale in Udine between 1 April 2024, and 30 September 2024. The inclusion criteria included evidence of ischemic stroke, in accordance with the World Health Organization (WHO) diagnostic criteria for stroke [20]. Patients were excluded based on the following criteria: (1) Patients unsuitable for BIA measurements due to hemodynamic instability, amputations, presence of implanted metal devices (such as pacemakers or metal prostheses), or skin lesions in the area of electrode application; (2) Missing data. (3) Patients transferred to Stroke Unit from ICU (Intensive Care Unit) or other departments; (4) Patients or their family members who have not provided informed consent (Figure 1).

Patient data were collected, including age, sex, cardiovascular risk factors, history of previous cerebrovascular events, and pre-stroke disability level. Clinical and therapeutic information related to acute ischemic stroke (AIS) was also documented, including baseline National Institutes of Health Stroke Scale (NIHSS) scores, details of recanalization therapies (such as intravenous thrombolysis and/or mechanical thrombectomy), and post-stroke disability, evaluated using the modified Rankin Scale (mRS) at 90 days. Weight and height were measured, and bioimpedance analysis was performed within 48 h of admission to the Stroke Unit. Laboratory blood tests were also evaluated, including absolute lymphocyte count, C-reactive protein, glycated hemoglobin, total proteins, albumin, total cholesterol, HDL, LDL, and triglycerides. The administrative health databases of the ASUFC in Udine were used. The data were anonymized but could be linked at the individual patient level through a unique stochastic key for each patient.

### 2.2. Bioelectrical Impedance Analysis

Within 48 h of admission to the Stroke Unit, patients were assessed using Bioelectrical Impedance Analysis (BIA) with the BIA 101 BIVA PRO (Akern^®^, Akern s.r.l., Pisa, Italy) device to determine R and Xc (Ω) values. Data on height and weight were also collected. For the Bioimpedance Analysis, electrodes were positioned using the hand-to-foot technique in accordance with recommendations [16].

The obtained R and Xc values were standardized by height in meters to calculate R/H and Xc/H. These values were plotted on the bioimpedance vector graph using the Hospital Bodygram software (version 3.0.33), referencing the healthy Italian population [19]. The R/H and Xc/H data for the patients were analyzed in relation to the 50%, 75%, and 95% tolerance ellipses of the reference population. Patients were classified as malnourished if their impedance vector fell within the lower or upper right quadrants and outside the 50% tolerance ellipse along the horizontal axis, as indicated in the literature [16,21].

### 2.3. Primary Outcomes

The primary outcome of the study was to compare the ordinal distribution of 90-day mRS scores between malnourished and non-malnourished patients based on the BIVA parameter [16]. The mRS assessment at 90 days was conducted by a stroke-specialized neurologist during a follow-up clinical visit. If a clinical visit was not feasible, the assessment was performed via a telephone interview.

### 2.4. Statistical Analysis

We applied the statistical technique of Inverse Probability Weighting (IPW) to balance the baseline characteristics of the cohorts, distinguishing between patients who were malnourished and those who were not at the time of admission to the Stroke Unit according to the BIVA method. This approach was employed to reduce the influence of confounding factors by estimating the probability of malnutrition presence or absence based on a set of relevant variables that could influence group assignment.

Initially, we calculated the probability of assignment to the malnutrition group by adjusting for a set of predefined covariates, known as the propensity score. The covariates included age, sex, presence of arterial hypertension, diabetes mellitus, coronary artery disease, atrial fibrillation, history of prior cerebrovascular events and/or malignancies, alcohol abuse, stroke etiology classified by TOAST criteria, use of intravenous thrombolysis and/or mechanical thrombectomy, pre-stroke mRS, and admission NIHSS. Subsequently, stabilized weights were calculated by dividing the raw probability of observed exposure by the propensity scores. The balance of weights was assessed using standardized mean differences (SMD), with differences less than 0.2 considered acceptable and those below 0.1 deemed negligible. These weights were then used to balance baseline covariates, generating a pseudo-population in which the influence of measured confounders was minimized, effectively simulating a condition of pseudo-randomization.

To compare laboratory data and BMI between malnourished and non-malnourished patients, we used Student’s *t*-test for parametric variables and the Wilcoxon-Mann-Whitney test for non-parametric variables. The normality of distribution was assessed using the Shapiro-Wilk test, with a significance threshold set at *p* < 0.05.

A weighted ordinal logistic regression (shift analysis) was used to evaluate the shift in mRS scores at 90 days in malnourished patients compared to those without malnutrition, considering prespecified clinical variables of interest.

Statistical analysis was performed using R Studio (version 2024.4).

## 3. Results

**Figure 2:**
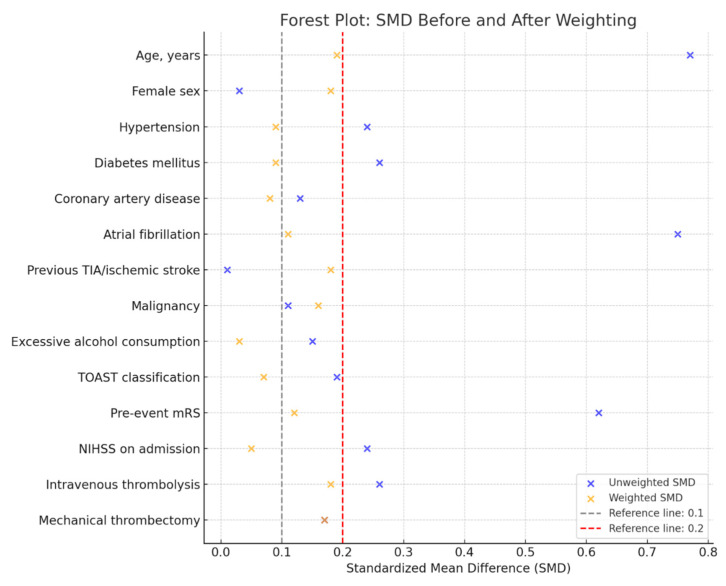
SMDs before and after weighting.

A total of 195 patients with ischemic stroke were analyzed using the BIVA method. Of these, 158 patients (81%) were not malnourished at the time of admission to the Stroke Unit, while 37 patients (19%) were classified as malnourished. The results, both weighted and unweighted for demographic, clinical, and medical history characteristics, are summarized in Table 1. Overall, a good balance was achieved for all major variables of interest, as shown in Figure 2.

At admission to the Stroke Unit, patients had a mean BMI of 26.28 ± 4.58, with a significant difference between the two groups (*p* = 0.021): BMI was significantly lower in malnourished patients (mean 24.82 ± 4.01) compared to non-malnourished patients (mean 26.62 ± 4.65).

Laboratory analyses performed at admission to the Stroke Unit, divided between malnourished and non-malnourished patients, are presented in Table 2. Statistically significant differences were observed in absolute lymphocyte counts, albumin concentration, and total and LDL cholesterol levels. In particular, malnourished patients, as identified by the BIVA parameter, had lower absolute levels of lymphocytes, albumin, total cholesterol, and LDL compared to other patients. These findings are consistent with evidence in the literature provided by major laboratory composite scores, such as the CONUT score and PNI score [6,9].

**Figure 3:**
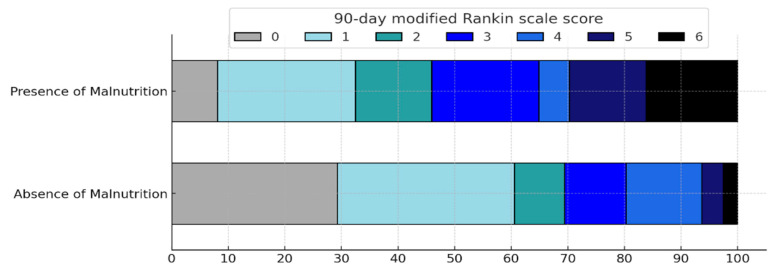
Distribution of modified Rankin Scale (mRS) scores at 90 days post-cerebrovascular event in patients with and without malnutrition.

The weighted ordinal logistic regression summarized in Table 3 and the shift analysis presented in Figure 3 reveal a significant difference in the ordinal distribution of mRS scores at 90 days post-ischemic cerebrovascular event. Malnourished patients, as determined by BIVA parameters, were over three times more likely to experience an mRS shift compared to non-malnourished patients (cOR 3.34, 95% CI 1.74–6.41; *p* = 0.001).

When considering the covariates of interest, malnutrition remained a predictor of poorer outcomes (acOR 2.79, 95% CI 1.37–5.70; *p* = 0.005), alongside baseline NIHSS (acOR 1.19, 95% CI 1.11–1.28; *p* < 0.001), use of intravenous thrombolysis (acOR 0.28, 95% CI 0.15–0.52; *p* < 0.001), absolute lymphocyte count (cOR 1.01, 95% CI 1.00–1.02; *p* = 0.027), and albumin concentration (cOR 0.82, 95% CI 0.75–0.89; *p* < 0.001).

## 4. Discussion

Our single-center observational study, based on an IPW analysis, revealed that the presence of malnutrition, identified through the BIVA method during the acute phase of ischemic stroke patients, is associated with worse outcomes, as evidenced by a less favorable distribution of mRS scores at 90 days.

The association between malnutrition and poor prognosis in stroke patients is well established in the literature. Traditionally, malnutrition has been assessed using composite laboratory-based scores [6,7,8,9]. The significance of these indices is supported by our findings, which demonstrate, through multivariate analysis, a strong correlation between clinical outcomes and lymphocyte and albumin levels measured at the time of admission to the Stroke Unit, as previously demonstrated in studies on the application of the CONUT score and the PNI score [6,9].

Although these composite laboratory scores are simple to apply and useful for detecting malnutrition and predicting long-term outcomes, they have certain limitations. They do not provide a real-time assessment of nutritional status and fluid balance, nor do they allow for continuous, precise monitoring over time as effectively as BIA/BIVA models. Additionally, the calculation of these scores relies on acquiring biochemical indicators through often invasive procedures, which are constrained by the diagnosis and treatment of the underlying condition, making them less practical for dynamic evaluation.

Consequently, there is a need to identify new indicators that enable a dynamic assessment of nutritional status during the acute phase of stroke and provide predictive value for prognosis.

In this context, BIA is now widely recognized as a valid tool for assessing nutritional status and predicting prognosis in patients with cancer, those undergoing hemodialysis, or those with heart failure [13,14,15]. Its widespread use is attributed to several key advantages: objective and reliable results, a non-invasive and patient-friendly methodology, ease of application, rapid assessment, and high reproducibility of measurements [22].

The application of the BIA/BIVA-based approach in stroke patients has been explored in only a limited number of studies to date. Most research has focused on the evaluation of patients in the post-acute phase, primarily in rehabilitative settings, often without distinguishing between ischemic and hemorrhagic stroke. These studies have mainly concentrated on analyzing the presence of sarcopenia and malnutrition in patients during the post-acute rehabilitative phase [23,24,25,26,27].

Researchers who have used bioimpedance analysis in acute stroke patients have primarily focused on phase angle (PhA) as a prognostic indicator [22,28,29,30]. A systematic review analyzed PhA in acute and subacute stroke patients, showing an inverse correlation between PhA and malnutrition, as well as reduced physical function [31]. PhA was significantly correlated with subjective and objective indicators of nutritional status, as well as sarcopenia and hydration [32].

However, compared to bivariate distribution models, PhA provides a less comprehensive analysis and lacks reliable, standardized cut-off values [17]. Therefore, comparisons of PhA values between different individuals should be limited to patient groups that are homogeneous in terms of sex, ethnicity, and BMI [17]. Indeed, patients with the same phase angle can present with very different conditions, ranging from malnutrition to a simple and isolated fluid overload. Thus, using phase angle alone as a prognostic indicator without standardizing it with other parameters could assign a worse prognosis to patients with fluid overload and a better prognosis to dehydrated patients [33]. In contrast, BIVA models allow for more sensitive bioelectrical measurements and provide more accurate information on nutritional and hydration status, making them suitable for analysis and comparison across even heterogeneous populations [17,34]. For these reasons, PhA values were not included in our study, and we opted for an analysis using bivariate distribution models. This approach has never been previously applied to patients with acute ischemic stroke.

The collected data highlight a statistically significant difference in body mass index (BMI) between the two groups, with lower values in malnourished individuals compared to non-malnourished individuals. This parameter was intentionally excluded from the regression analysis, as all patients were assessed using BIA, a much more precise tool for evaluating nutritional status and overall health than BMI. While BMI is simple and easy to calculate, it is often inaccurate and comes with several limitations. In contrast, bioimpedance analysis offers greater sensitivity and specificity, making BMI potentially outdated and insufficient for a comprehensive assessment [35,36].

The underlying causes of the relationship between malnutrition and a worse prognosis in patients with ischemic stroke are not yet fully understood. Multiple factors likely contribute to this association from both physiological and pathological perspectives. Firstly, inadequate nutritional intake may impair the body’s compensatory mechanisms, reducing its ability to maintain allostasis. In addition, malnutrition is often linked to systemic inflammation through a bidirectional relationship, increasing the risk of infection, cancer, and chronic diseases [37,38].

Our analysis has several strengths: (1) a prospective study design, (2) a systematic and structured data collection process, (3) the use of IPW analysis to minimize selection bias, and (4) an objective assessment of malnutrition, avoiding indirect measures such as scales or blood tests. However, there are some limitations to consider: (1) the study is single-center, and consequently, the available sample size is limited; (2) bioimpedance analysis was not always performed at the time of admission to the Stroke Unit but within 48 h of admission, which could have influenced body composition, especially in more severe patients unable to feed themselves independently; and (3) bioimpedance analysis was conducted in a semi-intensive setting like the Stroke Unit, attempting to minimize sources of electromagnetic artifacts, though these cannot be completely eliminated.

The adoption of different dietary regimes during hospitalization or stays in facilities other than the Stroke Unit may impact outcomes. Similarly, variations in outcomes may depend on the type of stroke, particularly in cases of hemorrhagic stroke, or on geographical and ethnic differences among the populations involved. Further studies will be needed to explore these variables in the future.

## 5. Conclusions

Malnutrition, assessed through BIVA at the time of admission to the Stroke Unit, is associated with worse clinical outcomes at 90 days following an ischemic cerebrovascular event.

Current literature highlights that the use of bioimpedance analysis in the management of cerebrovascular disease is primarily confined to rehabilitative settings. This study, however, emphasizes the importance of evaluating these parameters during the acute phase, as they provide essential information on the nutritional and hydration status of patients admitted to Stroke Units. These data are crucial for guiding therapeutic decisions, such as the administration of diuretics or fluids, and have significant prognostic value for clinical outcomes, as demonstrated by the results of this study.

In the future, early nutritional screening with BIVA in Stroke Units could prove to be an effective tool for promptly identifying malnourished patients, enabling targeted interventions with tailored supplements and diets. These strategies could help improve the health of hospitalized patients and optimize long-term outcomes.

## Figures and Tables

**Table 1:** Demographic and clinical characteristics (Legend: SMD = standardized mean difference; IQR = interquartile range; TIA = transient ischemic attack; TOAST = Trial of Org 10172 in Acute Stroke Treatment; mRS = modified Rankin Scale; NIHSS = National Institutes of Health Stroke Scale. † More than 1 liter of alcohol per day).

Variables	Overall Population (N = 195)	Presence of Malnutrition (N = 37)	Absence of Malnutrition (N = 158)	SMD Unweighted	SMD Weighted
Age, years [median (IQR)]	76 (65–82)	80 (75–86)	75 (63–82)	0.77	0.19
Female sex [n, (%)]	87 (44.6)	17 (45.9)	70 (44.3)	0.03	0.18
Hypertension [n, (%)]	148 (75.9)	31 (83.8)	117 (74.1)	0.24	0.09
Diabetes mellitus [n, (%)]	36 (18.5)	10 (27.0)	26 (16.4)	0.26	0.09
Coronary artery disease [n, (%)]	20 (10.2)	5 (13.5)	15 (9.5)	0.13	0.08
Atrial fibrillation [n, (%)]	61 (31.3)	22 (59.5)	39 (24.7)	0.75	0.11
Previous TIA/ischemic stroke [n, (%)]	26 (13.3)	5 (13.5)	21 (13.3)	0.01	0.18
Malignancy [n, (%)]	35 (17.9)	8 (21.6)	27 (17.1)	0.11	0.16
Excessive alcohol consumption ^†^ [n, (%)]	23 (11.8)	3 (8.1)	20 (12.7)	0.15	0.03
TOAST classification				0.19	0.07
Large artery atherosclerosis [n, (%)]	25 (12.8)	4 (10.8)	21 (13.3)		
Cardioembolism [n, (%)]	62 (31.8)	19 (51.3)	43 (27.2)		
Small vessel occlusion [n, (%)]	30 (15.4)	1 (2.7)	29 (18.3)		
Other determined cause [n, (%)]	4 (2.1)	0 (0.0)	4 (2.5)		
Undetermined cause [n, (%)]	74 (37.9)	13 (35.1)	61 (38.6)		
Pre-event mRS [median (IQR)]	0 (0–1)	1 (0–3)	0 (0–1)	0.62	0.12
NIHSS on admission [median (IQR)]	4 (2–9)	5 (2–13)	4 (2–8)	0.24	0.05
Intravenous thrombolysis [n, (%)]	89 (45.6)	13 (35.1)	76 (48.1)	0.26	0.18
Mechanical thrombectomy [n, (%)]	27 (13.8)	7 (18.9)	20 (12.7)	0.17	0.17

**Table 2:** Laboratory data (Legend: SMD = standardized mean difference; IQR = interquartile range; HbA1c = glycated hemoglobin; HDL = high-density lipoprotein; LDL = low-density lipoprotein). Bold indicates statistically significant results.

Variables	Overall Population (N = 195)	Presence of Malnutrition (N = 37)	Absence of Malnutrition (N = 158)	*p*
Lymphocytes, μL [median (IQR)]	1440 (1110–1820)	1210 (870–1720)	1490 (1157–1835)	**0.013**
C-reactive protein, mg/L [median (IQR)]	3 (1–11)	5 (1–14)	3 (1–10)	0.582
HbA1c, % [median (IQR)]	5.9 (5.6–6.2)	5.8 (5.6–6.1)	5.9 (5.6–6.2)	0.782
Total protein, g/L [median (IQR)]	65 (61–68)	64 (60–67)	65 (61–68)	0.311
Albumin, g/L [median (IQR)]	40 (37.7–42)	38 (36.7–40.2)	40 (38–42.2)	**0.007**
Total cholesterol, mg/dL [median (IQR)]	168 (135–204)	147 (122–159)	172 (138–211)	**0.001**
HDL, mg/dL [median (IQR)]	48 (42–57)	46 (43–59)	49 (40–57)	0.755
LDL, mg/dL [median (IQR)]	92 (67–125)	67 (53–92)	99 (74–128)	**0.001**
Triglycerides, mg/dL [median (IQR)]	91 (72–123)	88 (69–118)	91 (74–127)	0.171

**Table 3:** Weighted ordinal logistic regression according to the mRS scale at 90 days post-ischemic cerebrovascular event (Legend: mRS = modified Rankin Scale; NIHSS = National Institutes of Health Stroke Scale; IVT = intravenous thrombolysis; LDL = low-density lipoprotein). Bold indicates statistically significant results.

	mRS Shift (Univariate)		
*Predictors*	*Common Odds Ratio*	*CI*	*p*
Presence of malnutrition	3.34	1.74–6.41	**0.001**
	**mRS Shift (Multivariate)**		
*Predictors*	*Adjusted Common Odds Ratio*	*CI*	*p*
Presence of malnutrition	2.79	1.37–5.70	**0.005**
Female sex	0.85	0.48–1.51	0.580
NIHSS at admission (per unitary increase)	1.19	1.11–1.28	**<0.001**
IVT	0.28	0.15–0.52	**<0.001**
Mechanical thrombectomy	0.41	0.14–1.18	0.097
Lymphocytes (per unitary increase)	1.01	1.00–1.02	**0.027**
Albumin (per unitary increase)	0.82	0.75–0.89	**<0.001**
Total cholesterol (per unitary increase)	0.99	0.97–1.02	0.875
LDL (per unitary increase)	1.01	0.98–1.03	0.742

## Data Availability

The original contributions presented in the study are included in the article, further inquiries can be directed to the corresponding author.
